# Role of autopsy imaging‐computed tomography in the post‐mortem study of farm animals

**DOI:** 10.1002/vro2.1

**Published:** 2021-04-07

**Authors:** Kazutaka Yamada, Taiki Yokoyama, Naoyuki Aihara, Yumi Une, Reiichiro Sato

**Affiliations:** ^1^ School of Veterinary Medicine Azabu University Sagamihara Japan; ^2^ Veterinary Teaching Hospital Azabu University Sagamihara Japan; ^3^ Faculty of Veterinary Medicine Okayama University of Science Imabari Japan; ^4^ Faculty of Agriculture University of Miyazaki Miyazaki Japan

**Keywords:** diagnostics, imaging, radiology

## Abstract

**Background:**

Autopsy imaging (Ai) is used to determine the cause of death, providing pre‐dissection information. Ai is often used in the field of human forensic medicine but has never been applied on farm animals.

**Methods:**

Ai‐computed tomography (CT) was performed before necropsy for farm animals (one goat, one ox, one cow and three calves) that died or were euthanised.

**Results:**

Ai‐CT findings of rib fractures (case 1), urethral calculi (case 2), multiple osteolytic bone lesions (case 3 and 4) and hair balls (case 4) were confirmed by dissection. However, a tentative diagnosis of actinomycosis was made in an ox (case 5) using antemortem radiography and Ai‐CT, and the mass was identified as ameloblastic fibro‐odontoma on histological examination. A tentative diagnosis of maxillary abscess was made from antemortem radiography in a cow (case 6); however, the lesion was shown to be maxillary neoplasia on Ai‐CT. The mass was identified as hemangiosarcoma on histopathological examination.

**Conclusion:**

Ai is helpful in pathological examination because the specific findings are known before the dissection, the lesions can be pinpointed in the pathological dissection, facilitating workflow; furthermore, the oversight of lesions can be reduced. In addition, Ai‐CT images, including three‐dimensional images and a three‐dimensional printed model, allowed an easy understanding of pathology among students and farmers. Ai‐CT for farm animals represents a novel option for veterinary education.

## INTRODUCTION

Autopsy imaging (Ai) is used to determine the accurate cause of death, providing pre‐dissection information. The term post‐mortem imaging simply means imaging after death, which does not include pathological dissection. On the other hand, Ai provides imaging findings to guide in dissection, and feedbacking Ai again based on the findings of dissection plays a complementary role. In human medical practice, Ai is often performed in the field of forensic medicine.^1^ In veterinary medicine, Ai is currently seldom performed, due to the high costs and the unavailability of computed tomography (CT), which makes performing Ai before necropsy difficult. Therefore, only a few Ai reports have been published in the field of forensic veterinary medicine including in animal abuse^2^; in cases of a fox^3^ and a lynx^4^; and in cases of economically valuable animals such as a kangaroo,^5^ a thoroughbred,^6^ and a penguin.^7^ Ai has never been performed for farm animals with the aim of determining the cause of death for forensic reasons or confirmation of treatment. Farmers do not wish to pay much for the diagnosis and treatment of farm animals. Even more, there have been no idea of taking CT after death for farm animals. Therefore, to date, there was no report of Ai‐CT of farm animals. Here, we reported characteristic Ai‐CT findings and found a new role for Ai‐CT in farm animals through the Ai‐CT experience.

## MATERIALS AND METHODS

### Cases

Different farm animals (one goat, one ox, one cow and three calves) in this study were admitted to the Department of Large Animals Clinic, Veterinary Teaching Hospital, at Azabu University during the period 2017−2019. Ai‐CT was performed before necropsy for animals that died or were euthanised. Descriptions of the cases were shown in Table 1. The protocol of this study was approved by Guide for Animal Care and Use Committee at Azabu University, School of Veterinary Medicine (No. 160516‐10).

**TABLE 1:** Description of farm animals of autopsy imaging‐computed tomography

	Species	Sex	Age	Body weight (kg)
Case 1	Crossbred	Male	1‐month	63
Case 2	Crossbred‐goat	Cast‐male	2‐year	59
Case 3	Holstein Friesian	Female	5‐month	70
Case 4	Crossbred	Male	1‐month	64
Case 5	Japanese Black	Male	1‐year	179
Case 6	Holstein Friesian	Female	9‐year	674

### Computed tomography

Ai‐CT images were obtained by 80‐row multidetector helical CT scanner (Aquilion PrimeSP, Canon, Ohtawara, Japan). Cases 1−4 underwent whole‐body scan, cases 5 and 6 were too large to enter a CT gantry; therefore, the neck was cut, and Ai‐CT of only the head was performed. Imaging parameters were as follows: tube voltage, 120−135 kV; tube current, 200−400 mA; tube rotation time, 0.5−1.0 s/rotation; scanning slice thickness, 1.0−1.25 mm; and reconstruction slice thickness, 1.0−5.0 mm. Digital Imaging and Communications in Medicine (DICOM) data were sent to a viewer (Newton OsiriX, Newton‐Graphics, Sapporo, Japan) to allow the evaluation of images in the reconstructed bone, lung, and soft‐tissue algorithms, and fixed volume rendering (VR) and maximum intensity projection images.

### 3D printer

DICOM data of case 5 were transferred to a three‐dimensional (3D) printer unit (Moment 220, Richo, Tokyo, Japan), and the bones of the skull model were printed out at half‐scale.

## RESULTS

### Case 1

**FIGURE 1:**
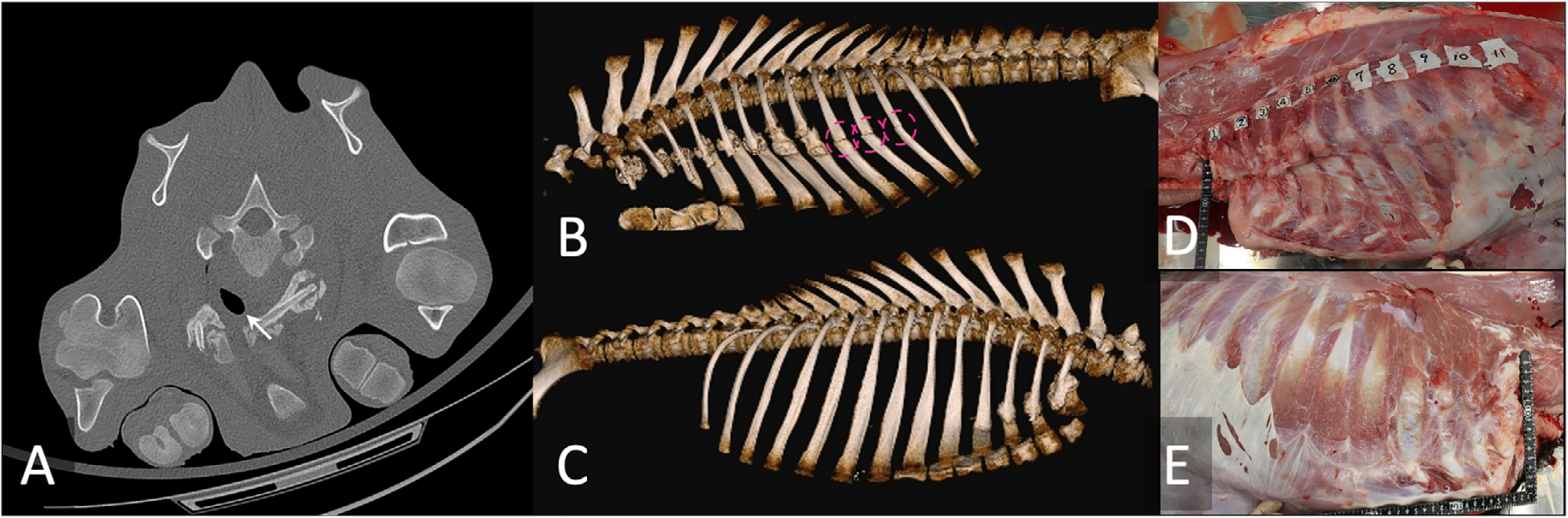
Autopsy imaging‐computed tomographic images in case 1. (A) Two‐dimensional image at a level of the first rib; (B) Three‐dimensional image of the left‐side view; (C) Three‐dimensional image of the right‐side view; (D) Gross photograph of the left‐side view, (E) Gross photograph of the right‐side view. The left fractured rib dislocated the tracheal stenosis (A, arrow). There were cracked ribs with no bony callus in ninth to eleventh ribs (B, circles). Fractures were confirmed on necropsy (D, E)

A 1‐month‐old, male cross‐bred calf showed respiratory difficulty, including wheezing, coughing, tachypnea and abnormal chest sounds. In the antemortem standing radiograph, fractures in the fifth, sixth, seventh and eighth ribs were depicted. Despite medical treatment, the clinical condition did not improve, and the calf was judged to have a poor prognosis and was thus humanely euthanised. On Ai‐CT, the first left rib was observed to be fractured, and it had dislocated the tracheal stenosis (Figure 1A). Whether the rib fractures were in the right or left sides could not be distinguished in the antemortem standing radiograph. However, in the Ai‐CT, it was easily determined that the fractures were on both sides, with fractures in the first to eleventh ribs on the left side and fractures in the first to fourth ribs on the right side (Figure 1B,C). In addition, Ai‐CT VR images (Figure 1B and C) demonstrate an interpretable evidence of the fractures. Fractures were confirmed by necropsy (Figure 1D,E ).

### Case 2

**FIGURE 2:**
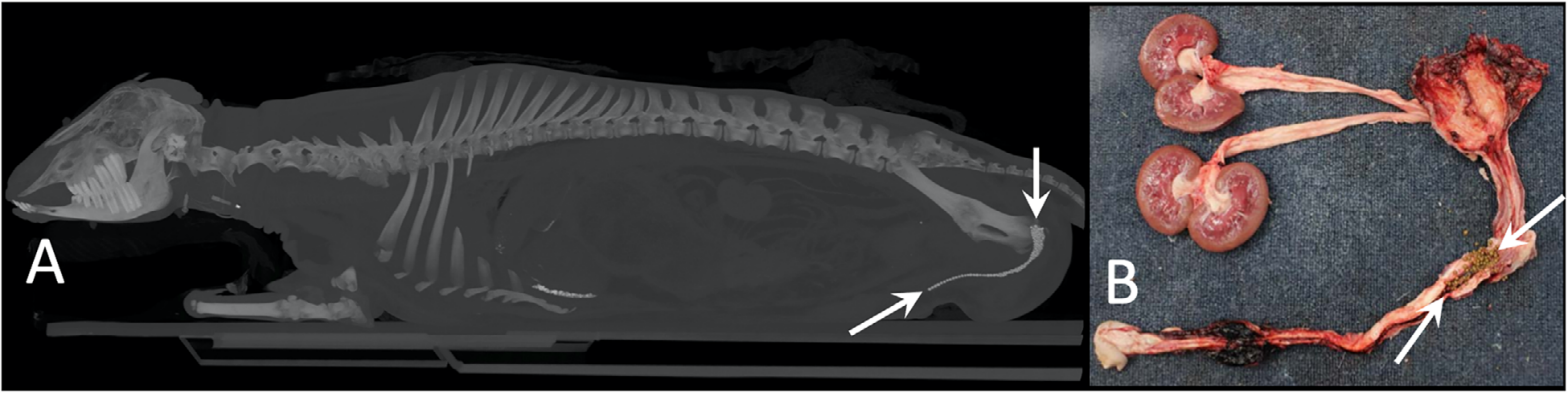
Autopsy imaging‐computed tomographic maximum intensity projection image (A) and gross photograph (B) in case 2. Arrows show the urethral calculi. Sandy contents in the reticulum were also depicted as high density in the cranial ventral abdomen

A 2‐year‐old, castrated male cross‐bred goat showed dysuria. This case had a history of cystectomy. An antemortem radiograph revealed urinary bladder calculi but no urethral calculi. It was a case of sudden death, and Ai‐CT was performed the same day. The urethral calculi were depicted on Ai‐CT (Figure 2A), and peritoneal fluid and free gas were also observed in the abdomen. The urethral calculi and bladder rupture were confirmed by necropsy (Figure 2B).

### Case 3

**FIGURE 3:**
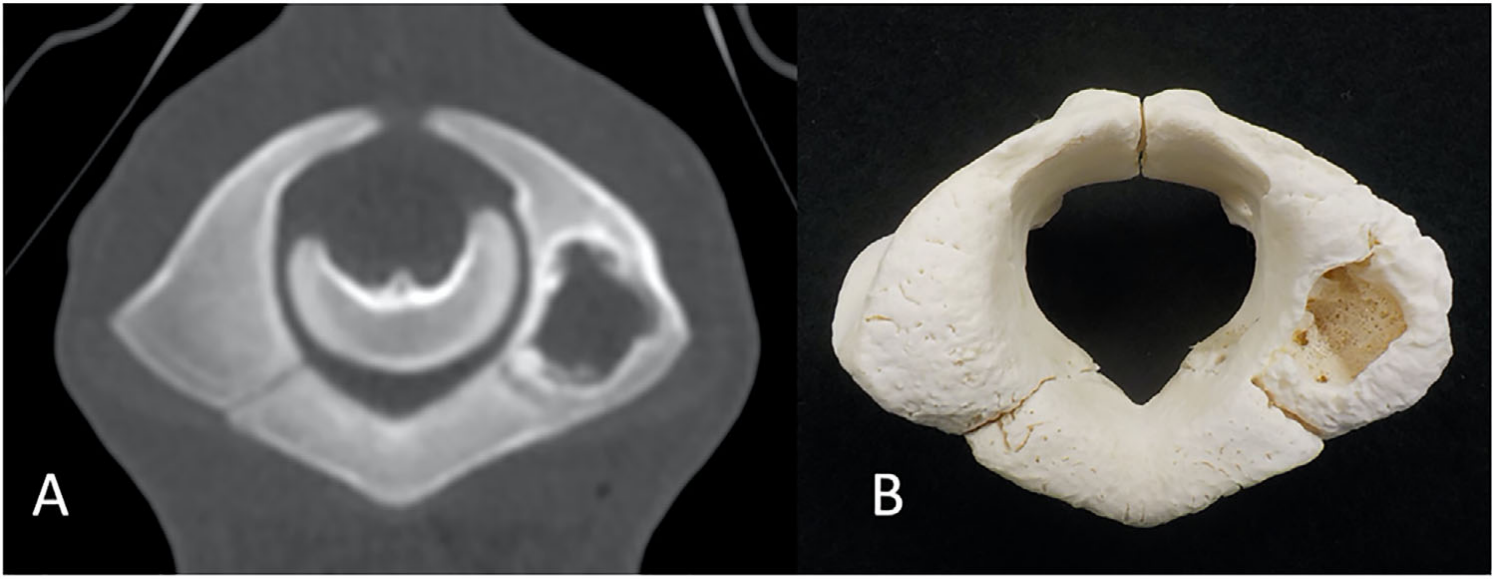
Autopsy imaging‐computed tomographic image (A) and gross photograph (B) in case 3. Bone lysis in the atlas (A), which is one of multiple bone lysis lesions, was confirmed (B)

A 5‐month‐old, female Holstein Friesian calf showed astasia 2 months prior. An antemortem radiograph did not reveal any abnormalities in the skull, spine, or limbs. Without determining the clinical diagnosis, the calf was considered to have a poor prognosis and was humanely euthanised. On Ai‐CT, multiple osteolytic bone lesions were observed in the occipital condyle; atlas (Figure 3A); fourth cervical spine; scapula; seventh, tenth and thirteenth thoracic spine; first rib; and distal femur. These osteolytic bone lesions were confirmed upon dissection (Figure 3B). Swab samples were obtained from the osteolytic lesions, and *Trueperella pyogenes* and *Fusobacterium necrophorum* were identified on bacterial culture.

### Case 4

**FIGURE 4:**
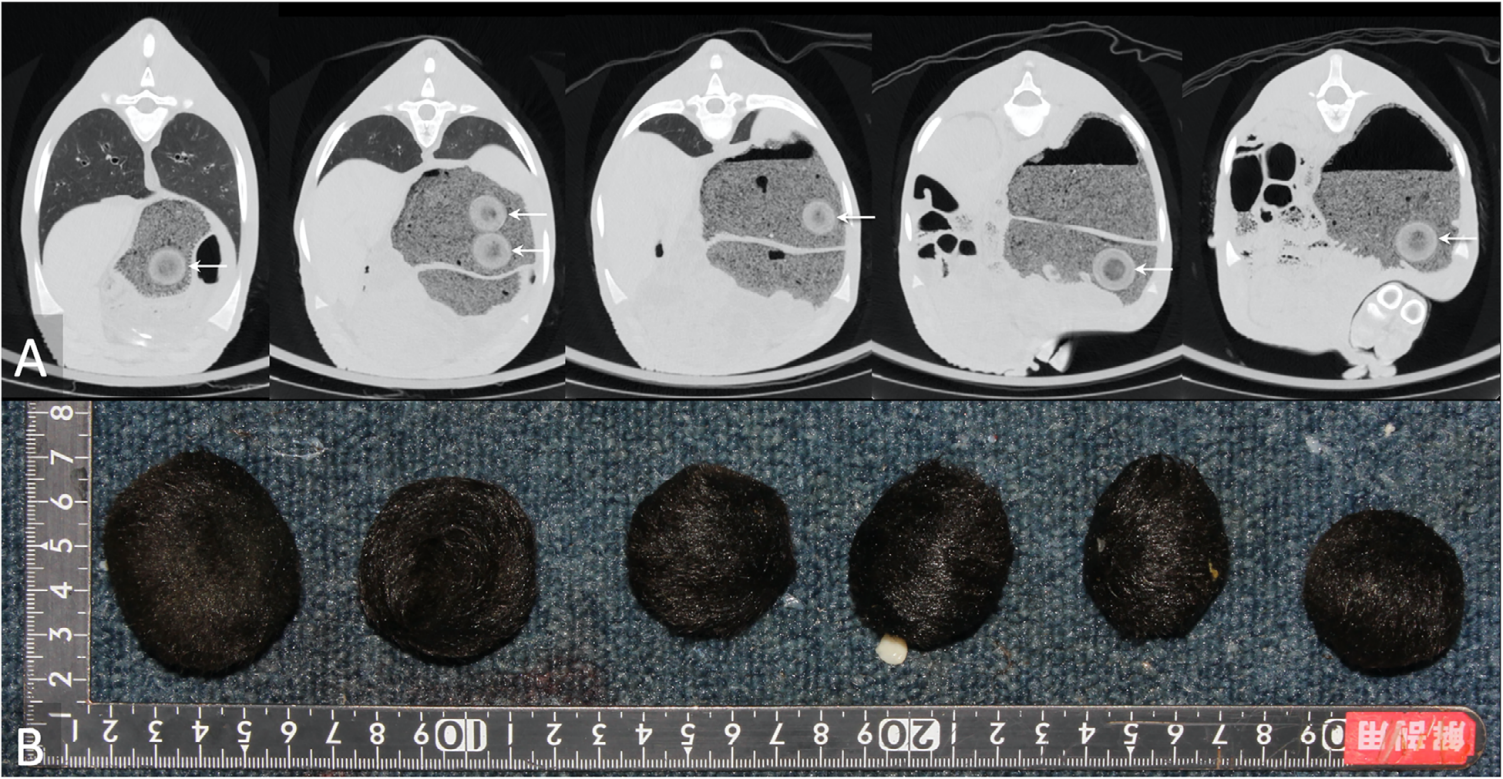
Autopsy imaging‐computed tomographic image (A) and gross photograph (B) in case 4. A total of six hairballs were depicted in the rumen (arrows), and all hairballs were confirmed on necropsy (B)

A 1‐month‐old, male cross‐bred calf showed astasia. Radiography was not performed in this case. The calf was judged to have a poor prognosis and was humanely euthanised. On Ai‐CT, multiple osteolytic bone lesions were found in the occipital condyle; atlas; axis; third to seventh cervical spine; scapula; fifth thoracic spine; eighth, ninth and thirteenth ribs; first, second, fifth and sixth lumbar spine; pelvis; distal femur; and proximal tibia. The osteolytic bone lesions were confirmed upon dissection. Swab samples were obtained from these lesions, and *Proteus mirabilis*, *Escherichia coli*, *Streptococcus uberis* and *F. necrophorum* were identified on bacterial culture. In addition, six spherical concentric‐layer foreign bodies with a diameter of 32−44 mm were detected on Ai‐CT (Figure 4A). Six hairballs in the rumen were confirmed upon dissection (Figure 4B).

### Case 5

**FIGURE 5:**
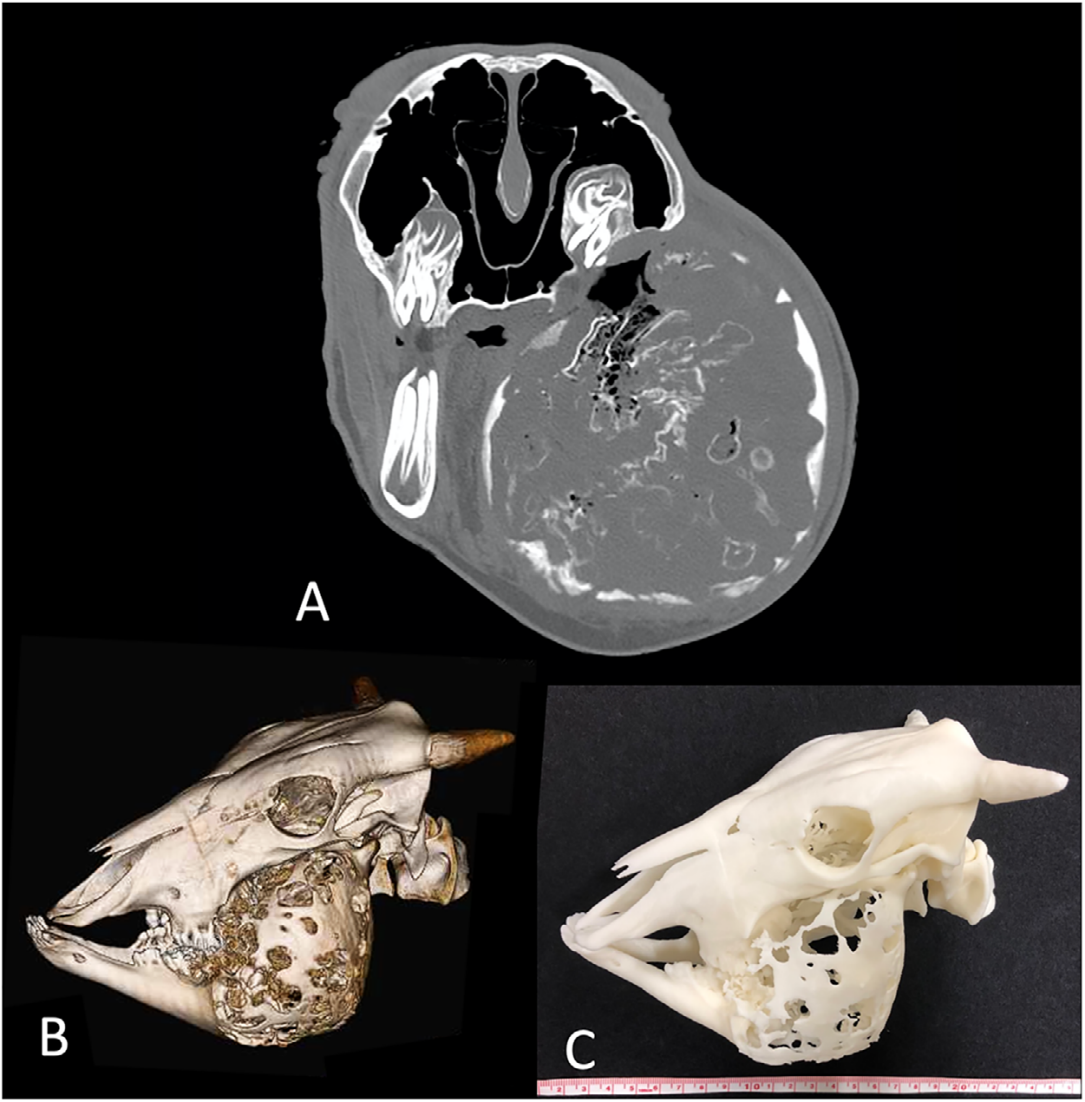
Autopsy imaging‐computed tomographic image in case 5. (A) Two‐dimensional image; (B) Three‐dimensional image; (C) Photograph of the three‐dimensional printed model). The use of the three‐dimensional printed model allowed the bony changes to be easy to understand making it ideal for hands‐on training

**FIGURE 6:**
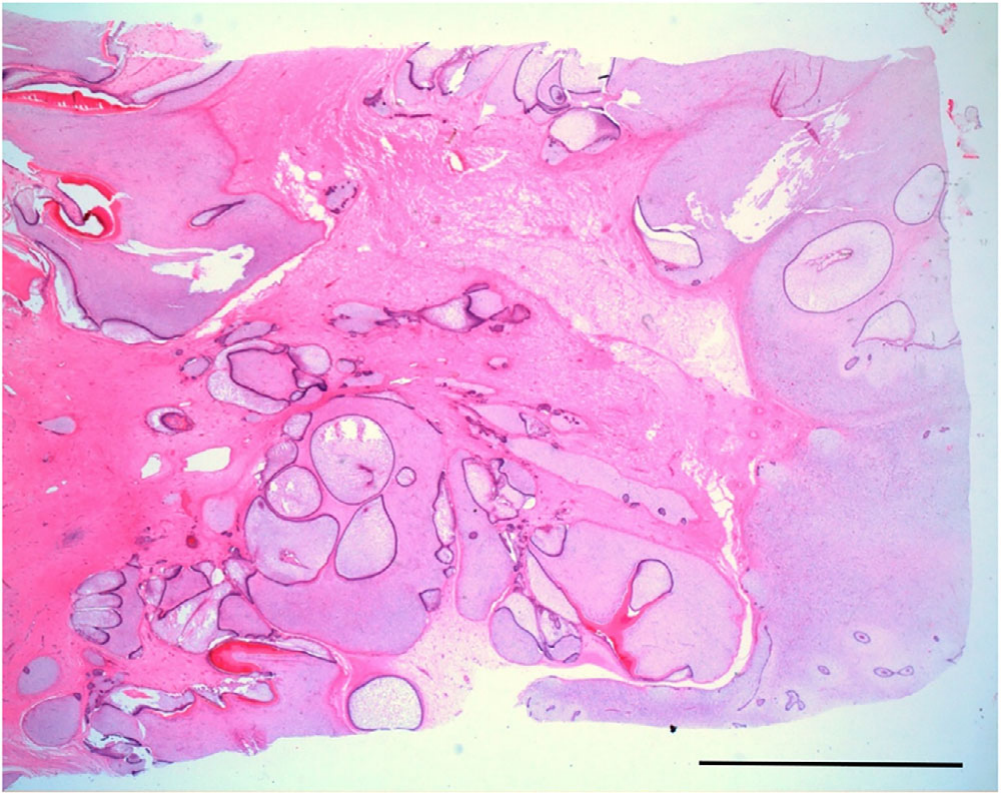
Histological findings in case 5. The neoplasia comprised various odontogenic tissues. (Hematoxylin and eosin stain. Bar, 10 mm). The mass was identified as ameloblastic fibro‐odontoma

A 3‐year‐old, male Japanese Black ox showed a mandibular swelling. An antemortem radiograph showed periosteal reaction and moth‐eaten osteolysis in the mandible, with swelling of the surrounding soft tissue. At that time, the tentative diagnosis was actinomycosis. The ox had been showing feeding difficulties; therefore, it was judged to have a poor prognosis and was humanely euthanised. A round‐shaped bony proliferation and osseous densities in the mass were depicted on Ai‐CT (Figure 5A and B), and the top differential diagnosis on the basis of Ai‐CT findings was actinomycosis. The 3D‐printed model demonstrated detailed bony changes (Figure 5C). On histopathological examination, the neoplasia comprised various odontogenic tissues (Figure 6). Tooth germ‐like tissue and enamel epithelium‐like cells were observed in the neoplasia. The mass was identified as ameloblastic fibro‐odontoma.

### Case 6

**FIGURE 7:**
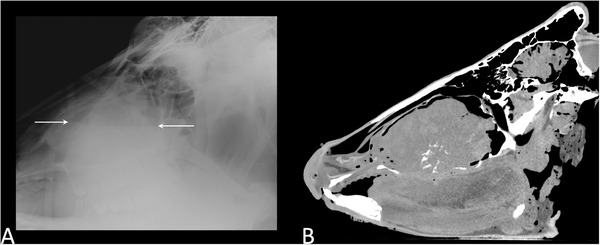
Antemortem radiograph of the skull (A) and autopsy imaging‐computed tomographic image (B) in case 6. Fluid level (arrows) in the antemortem radiograph (A) suggests maxillary abscess; however, aggressive neoplasia with calcification was detected in the autopsy imaging‐computed tomography (B). Intracranial air is a technical artifact because of the cutting of the neck. Air entered to subarachnoid space from the cutting site

**FIGURE 8:**
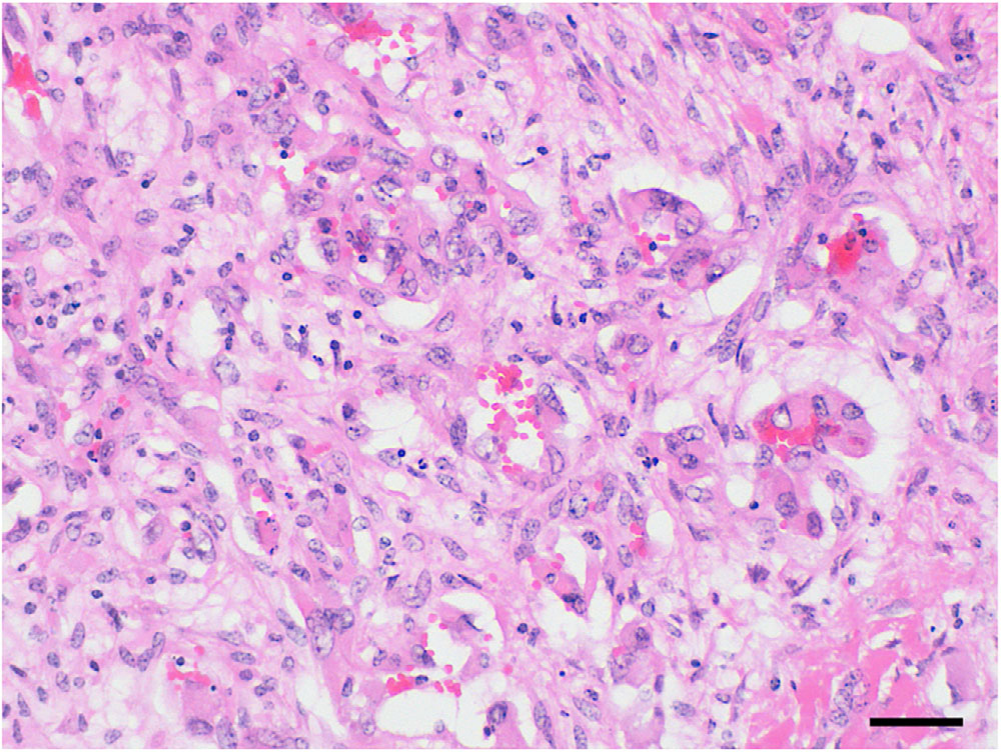
Histological findings in case 6. Neoplastic endothelial cells form irregularly shaped and sized vessels. (Hematoxylin and eosin stain. Bar, 20 μm). The mass was identified as hemangiosarcoma

A 9‐year‐old female Holstein Friesian cow showed a maxillary swelling. An antemortem radiograph showed a fluid level in the maxilla (Figure 7A), and the tentative diagnosis was a maxillary abscess. The animal suddenly died the day after radiography, and Ai‐CT was performed; it showed maxillary neoplasia with calcification (Figure 7B). On histopathological examination, the neoplasia comprised numerous irregularly shaped and sized vascular clefts, lined by plump or spindle‐shaped neoplastic cells (Figure 8). The fibrous stroma between the clefts was abundant, and large areas of necrosis and haemorrhage were observed in the neoplasia. The neoplastic cells were positive for Factor VIII‐related antigen. The mass was identified as hemangiosarcoma.

## DISCUSSION

In case 1, the fractured first left rib had displaced the tracheal stenosis; thus, wheezing was considered to be due to the physical compression of the trachea.^8^ The cranial thorax can be difficult to depict on a standing radiograph because the cranial thorax is superimposed with the scapula/humerus; however, CT images are cross‐sectional, allowing the cranial thorax to be observed easily.^9^ Furthermore, cracks with no bony callus were identified in the left ninth, tenth and eleventh ribs. Cracked ribs without callus, which are not thick, could not be recognised in the radiograph, even in retrospective observation, yet the fractured ribs that are thick with callus were able to be recognised by the radiograph. Based on differences in the fracture healing stages, the fractures in the ninth to eleventh ribs and those in the first to eighth ribs may have occurred at different times.^10^ Acute fractures exhibit sharply demarcated fracture margins on CT images, and chronic or healed factures usually exhibit a productive bridging.^11^ In the case of small animals, these findings suggest continuous animal abuse, with Ai playing an important role in forensic examinations. There was no episode of suffering rib fractures, the cause of fractures in this case were remained unknown. In the authors' opinion, it is unlikely that the farmers' calves were abused. The chronic fracture in cranial thorax may be injured due to failure of delivery assistance. The acute fracture in caudal thorax may be injured due to bad management, for example, slippery floors of extensive breeding in a small area. However, the ribs with bridged fractures are easily identified on necropsy, and nonbridging cracks are sometimes difficult to identify by dissection. If cracks are found prior to dissection, the pathologist can pinpoint the cracked ribs; this facilitates the workflow and may reduce the oversight of lesions. During spontaneous breathing CT examination, an artefact, which can occur due to respiratory motion,^12^ is sometimes mistaken as a rib fracture.^9^ Because a cadaver does not breathe, radiologists need not be concerned about motion artefacts on Ai images. Authors previously reported the utility of 3D images for teaching image interpretation to students.^13^ When explaining the pathology to the farmers, showing photographs of dissection is not considered acceptable because they are grotesque. However, Ai‐CT VR images (Figure 1B,C) can provide easily interpretable evidence to farmers. If the cause of the fracture is due to some failure of management by the farmer; e.g. a failure of delivery assistance, slippery floors or extensive breeding in a small area, providing the image interpretation can lead to improvements in management practices. The instruction using Ai hopefully contributes to increased productivity.

In case 2, the antemortem radiograph revealed urinary bladder calculi but not urethral calculi. The urethral calculi were depicted on Ai‐CT and were confirmed by necropsy. It was suspected that the calculi most likely moved from the urinary bladder to the urethra, and the sudden obstruction of the urethra resulted in urinary bladder rupture and the leakage of urine into the abdomen. The information regarding the site and the numbers of calculi resulted in a smooth dissection. Although free air in the abdomen cannot be confirmed by dissection, the detection of free gas is considered an advantage of Ai‐CT over necropsy. Urinary calculus formation has been associated with the dietary intake of minerals. Phosphorous‐rich diets have been most commonly associated with urolithiasis.^14^ The authors do not have any information about what feeding intake in this case. If this case was given inappropriate feed, showing interpretable Ai‐CT images to farmers is more convincing than explaining urolithiasis in word, resulting in improvements in farm management.

In case 3, the osteolytic bone lesion could not be depicted on radiographs because the lesions were blotted out by the thick trunk. Therefore, the depiction of the tiny lysis in the antemortem radiograph can be difficult. A complete detection of all multiple osteolytic bone lesions in dissection is also quite difficult; however, Ai‐CT allows these lesions to be detected. As demonstrated in case 1, Ai‐CT is useful for detecting bony changes. In addition, it takes a long time and more labour to fix bone specimens (Figure 3B), but Ai‐CT allows the non‐destructive observation of bony changes. However, the cause of multiple osteolytic bone lesion in this case were remained unknown even in pathological examination, it is certainly that there was a history of infection. It is authors' speculation that there was an umbilical cord infection after birth. Although the navel cord has healed, it is highly possible that the bacteria were distributed throughout the body via systemic blood flow.

In case 4, the number of hairballs in the rumen had already been identified before dissection; therefore, further exploration was not required for the pathologist, and this might have contributed to the shortened necropsy time. Although we have not usually found hairballs to be clinically significant in our experience, but sometimes they cause small intestine obstruction. One of the causes of hairballs is associated with the persistent sucking of penmates.^15^ The authors do not have keeping environment information in this case. If this case was kept group breeding, showing the image interpretation of hairballs provides information to farmers regarding optimal calves' management. In addition, showing the Ai‐CT findings of hairballs to veterinary students before dissection intrigued them into performing the pathological examination.

In case 5, the tentative diagnosis based on the antemortem radiograph findings was actinomycosis, and the top differential diagnosis based on the Ai‐CT findings was still most likely actinomycosis.^16^ But the mass was identified as an ameloblastic fibro‐odontoma on histopathological examination. Therefore, histopathological examination is necessary for definitive diagnosis. Radiographic appearance of bovine ameloblastic fibro‐odontoma has been previously reported,^17^ but the CT appearance reported in the present study has not been reported before. The 3D model, which was printed out at half‐scale, is smaller than the real size and not dirty; therefore, it is easier to handle. However, some limitations remain such as the availability and time‐consuming nature of 3D printing. Nevertheless, if possible, the use of a 3D model before dissection in the near future would provide case‐specific anatomical information and facilitating the understanding of the pathological condition to pathologists and students. Furthermore, 3D printer could be used to provide hands‐on training for veterinary students. The concept of applying 3D printers in veterinary clinics has been introduced previously,^18^ and an Ai‐CT 3D model may contribute to veterinary education.

In case 6, the tentative diagnosis based on antemortem radiograph findings was a maxillary abscess.^19^ The neoplasia, which could not be diagnosed antemortem, was diagnosed on Ai‐CT performed before necropsy. The neoplasia findings observed on Ai‐CT before dissection could provide useful information to pathologists. The mass was identified as hemangiosarcoma on histopathological examination. Bovine hemangiosarcoma is rare but has been previously reported,^20^ and to the authors’ knowledge, this is the first report of maxillary hemangiosarcoma.

In cases 3 and 4, osteomyelitis occurred as a result of a bacterial or fungal infection.^21^ *Salmonella dublin* was reportedly isolated from osteomyelitis lesions in calves,^22^ whereas *T. pyogenes*, *F. necrophorum*, *P. mirabilis*, *E. coli* and *S. uberis* were isolated from the calves in the present study. Thus, bacterial culture examination is necessary for a definitive diagnosis and to determine whether bone lysis was caused by bacteria or fungi. In cases 5 and 6, CT roughly helped determine whether a swollen face was caused by trauma, infection, or neoplasia; however, histopathological examination remains necessary for identifying neoplasia. Ai and bacterial culture/histopathological examinations complement each other when determining the cause of death.

In small animal cases, dissection can be difficult to perform because it is hard to obtain permission from the clients. In contrast, in farm animals, dissection is not difficult. Therefore, the advantage of Ai‐CT in the case of farm animals is that the imaging findings can be supported by necropsy. However, the limitation is the sizes of the animals for Ai‐CT. The CT used in this study, which was originally manufactured for humans, has a 72‐cm gantry diameter. Whole‐body scans were performed for cases 1–4, in which the body weights were under 70 kg, whereas the severed heads were scanned by CT in cases 5 and 6. Currently, a 100‐kg weight limit exists for performing Ai‐CT for farm animals in our experience.

There are case reports of cattle in antemortem CT examination,^16^, ^23^, ^24^ and there is no doubt about its use for clinical diagnosis. However, such processes are for clinical diagnosis and are expensive for farm animals. The diagnosis often does not pay for the cost and effort. The focus of this study is Ai before dissection as an academic research, not clinical service. Ai‐CT for farm animals plays an important role in guiding and providing information not only for the pathologists but also for the students and farmers. CT has so far not been performed before necropsy for farm animals after their death. Incorporating Ai‐CT before the pathological examination at a veterinary university can represent a novel option for veterinary education.

## CONCLUSION

Ai is helpful in pathological examination because the specific findings are known before the dissection and the lesions can be pinpointed in the pathological dissection, facilitating workflow. Furthermore, the oversight of the lesions can be reduced. In addition, Ai‐CT images, including 3D images and 3D printed model, allowed an easy understanding of pathology among the students and farmers. Ai‐CT for farm animals represents a novel option for veterinary education.

## CONFLICT OF INTEREST

The authors declare no conflict of interest.
